# A collaborative taxonomy of social media indicators for localised disaster response

**DOI:** 10.4102/jamba.v17i2.1839

**Published:** 2025-10-15

**Authors:** Priscila Carvalho, Zainab Akhtar, Manta Nowbuth, Yaw A. Boafo, Ebenezer F. Amankwaa, Catalina Spataru, Ferda Ofli, Muhammad Imran

**Affiliations:** 1 Energy Institute, Bartlett School of Environment, Energy and Resources, University College London, London, United Kingdom; 2 Qatar Computing Research Institute, Doha, Qatar; 3 Department of Civil Engineering, University of Mauritius, Reduit, Mauritius; 4 Centre for Climate Change and Sustainability Studies, University of Ghana, Accra, Ghana; 5 Department of Geography and Resource Development, University of Ghana, Accra, Ghana

**Keywords:** disaster response, social media, indicators, information needs, taxonomy, AI

## Abstract

Effective disaster management hinges on prompt, informed decisions, where social media has emerged as a real-time information source. However, current artificial intelligence (AI) systems for disaster response rely on universal taxonomies that assume information relevance is consistent across geographical and cultural contexts – an assumption that fails to account for regional variations in disaster types, response capabilities and local priorities. This study questions the ‘one-size-fits-all’ approach by developing context-specific social media indicator taxonomies through participatory engagement with 104 stakeholders across Ghana and Mauritius. We developed a taxonomy of 39 social media indicators across four categories: urgent needs, impact assessment, situational awareness and vulnerable populations. Our findings reveal significant regional variations in disaster information priorities that contradict assumptions underlying existing universal frameworks. While impact assessment indicators showed convergence between countries, other categories revealed that there are still important areas for future research on incorporating local stakeholder knowledge into AI system design. Our participatory methodology provides a replicable framework for developing adaptive, context-aware machine learning classifiers that can transform static universal categorisations into dynamic systems aligned with unique regional priorities and operational contexts.

**Contribution:**

We suggest future research areas that span across developing transfer learning approaches that leverage pre-trained multilingual models while incorporating region-specific context, creating active learning frameworks with local validation loops, implementing feedback mechanisms and establishing fair human-in-the-loop annotation processes that maintain quality.

## Introduction

Disasters, both natural and man-made, can have devastating consequences by causing widespread destruction and disruption in terms of livelihoods, infrastructure damage, biodiversity and livestock loss. Effective disaster management requires prompt, informed decisions, where social media has emerged as a real-time information source. Growing research demonstrates that social media networks serve as highly effective channels for rapid information distribution during emergencies, substantially improving situational awareness in disaster scenarios (Gao, Barbier & Goolsby 2011; Imran et al. 2014, 2015; Kumar et al. 2011). Platforms such as the former Twitter, WhatsApp and Facebook are increasingly being used by the public, where emergency managers are utilising information posted by locals in real time during a crisis event to enhance their crisis management capabilities (Purohit et al. 2014). Specifically, the information is used to monitor disaster events in real time, resolve rumours and misinformation, issue warnings and share public information (Abdul Mueez, Mardiana & Rosliza 2019). An example of resolving misinformation was evident during the 2018 Dead Sea flash flood disaster that hit Jordan, where a dam supervisor posted pictures and status updates on Facebook addressing rumours about the risk of flooding and dam conditions (Banikalef, Al Bataineh & Atoum 2018). Along with this, various social media monitoring and analysis tools have been developed and used by agencies and organisations to help manage and respond to disasters. Ushahidi (Okolloh 2009), for example, is designed to collect and visualise disaster reports, whereas Sahana Eden is tailored for relief and emergency response management (Duc, Vu & Ban 2014). Other tools include Geofeedia, Tweet Deck, Hootsuite and Facebook Crisis Response.

The inherent noise of social media data and its high volume generated during a crisis make manual extraction of information impossible. Filtering through vast amounts of data in a timely and efficient manner is a challenge being addressed by supervised machine learning models for text classification. CrisisDPS (Alam, Ofli & Imran 2019) is a ready-to-use data processing service with three crisis-relevant tweet classifications: (1) categorisation of disaster type, (2) informativeness categorisation and (3) categorisation of different types of humanitarian information. These services have been derived by combining seven publicly available humanitarian data sets (AIDR, CrisisLex, CrisisMMD, CrisisNLP, Disaster Response Data, Disasters on Social Media, and SWDM) After conducting extensive classification experiments using both classical and deep learning algorithms (i.e. Convolutional Neural Networks, Support Vector Machines and Random Forests, to name a few), the best models are used to support disaster response. Notably, these classifiers are designed to be universally applicable, irrespective of disaster types, geographic locations or information requirements of stakeholders managing or assessing disasters on the ground. CrisisLex (Olteanu et al. 2014) provides a lexicon for collecting and filtering crisis-related communications, categorising tweets into broad information type classes such as ‘Infrastructure Damage and Utilities’ and ‘Caution and Advice’. EMTerms (Temnikova, Castillo & Vieweg 2015) offers standardised terminology for crisis tweets, while CrisisNLP (Imran, Mitra & Castillo 2016) focuses on linguistic processing of crisis messages. Artificial intelligence for disaster response (AIDR) enables automatic classification of crisis tweets into predefined categories, and CrisisDPS combines multiple humanitarian data sets to provide universal classification services.

Research demonstrates that different user demographics use platform mechanisms differently, with users from different countries tending to use former Twitter differently – Germans used hashtags more often (suggesting a focus on information sharing), while Koreans tended to reply more often to each other (suggesting a focus on conversations) (Hong, Convertino & Chi 2011). Moreover, the characteristics of crisis events leave a distinctive ‘print’ on social media with respect to time and duration, including variations in the kind of information being posted and by whom (Hong et al. 2011), indicating that information relevance varies significantly across cultural and geographical contexts. Taxonomy’s universal approach assumes that information relevance is consistent across different geographical and cultural contexts – an assumption that fails to account for varying disaster types, response capabilities and local priorities. For instance, EMTerms (Temnikova et al. 2015) offers standardised terminology for crisis tweets but was developed through expert consultation without direct input from affected communities or local responders. While EMTerms provides more granular categories than CrisisLex, its top–down approach means it may miss critical information types that are relevant only in specific regional contexts. Neither EMTerms nor CrisisLex adequately captures information about traditional community support systems, local infrastructure peculiarities or region-specific vulnerable populations that may be crucial in non-Western disaster contexts. TREC-IS (McCreadie, Buntain & Soboroff 2020) represents progress with its 25 information type categories developed in consultation with emergency managers, yet it still reflects primarily North American and European perspectives. The CrisisMMD: Multimodal Crisis Dataset (Alam, Ofli & Imran 2018) offers humanitarian-specific categories that represent an advancement in crisis informatics by providing three types of annotations that include ‘Informative vs Not Informative’, ‘Humanitarian Categories’, and ‘Damage Severity Assessement’.

These taxonomies share three fundamental limitations. Firstly, the assumption of universality is problematic as disasters affect populations asymmetrically based on existing social inequalities, and recovery processes are highly contingent on local socio-economic conditions (Madianou 2015). Secondly, these taxonomies were developed primarily using data from Western countries and English-language sources, limiting their relevance for disaster response in many locations with non-English languages and the Global South. The predominant focus on English tweets in crisis research – with 97% of reviewed studies analysing only English-language data – overlooks the critical need for cross-language domain crisis response systems, particularly in non-English speaking countries where users post content in their local languages (Wahid et al. 2022). Moreover, the geographic bias is evident in data collection patterns, as exemplified by the stark contrast between 230 000 tweets collected during Typhoon Haiyan in the Philippines versus 4 million during Hurricane Sandy in the United States, reflecting underlying inequalities in digital infrastructure and representation (Crawford & Finn 2015). Thirdly, they lack mechanisms for incorporating local knowledge and priorities, which are crucial for effective disaster response in specific regions. Current approaches often fail to account for the ‘use case-dependent actionability’ of information, where what constitutes relevant or actionable information varies significantly based on the specific role of the user and the local context of the disaster (Kruspe, Kersten & Klan 2019). This limitation is particularly concerning given that emergency management agencies require flexibility and customisation of social media filtering and analysis algorithms to support responses in various specific contexts, rather than relying on pre-trained general-purpose models with limited generalisation capability.

This study explores a different approach by developing context-specific taxonomies through participatory engagement with local stakeholders in Ghana and Mauritius. Our methodology grounds categories in local realities by conducting extensive reviews of humanitarian documents and engaging 104 stakeholders across both nations to identify and prioritise information categories. Our results reveal substantial differences in priority indicators between countries – for example, Ghana prioritises ‘Children’ as the top vulnerable population indicator at 4.58/5, while Mauritius rates it at only 1.85/5. Our participatory methodology makes key contributions. Firstly, we demonstrate methodological innovation by showing how participatory approaches can address the limitations of universal taxonomies by incorporating local knowledge and priorities. Secondly, we provide empirical evidence of significant regional variations in disaster information priorities, challenging assumptions of universal applicability that underpin existing systems. Thirdly, we discuss a technical framework for developing adaptive, context-aware machine learning classifiers considering more bottom–up participatory taxonomies. Our approach not only promises to improve the relevance of AI-based disaster response systems but also addresses calls in the literature for more qualitative, context-sensitive approaches to crisis management through digitalisation in the Global South (Crawford & Finn 2015; Madianou 2015).

By considering future research pathways for integrating insights from stakeholders directly into the development of machine learning models, our research discusses a framework to train machine learning text classifiers to support systems alignment with the unique information requirements and priorities of each specific location. The proposed research area moves beyond static, universal categorisations to create dynamic, context-aware classification systems that can capture the unique priorities of each context while ensuring that disaster response is more effective and contextually appropriate. The remainder of this study presents our methodology, findings and implications for future research in developing localised artificial intelligence (AI) systems for disaster response. We underscore the need for disaster information systems to work with tailored taxonomy approaches that reflect the unique landscapes and communities they aim to serve, eschewing a generic, one-size-fits-all strategy commonly used today.

### Methodology for taxonomy development with stakeholder input

Our approach to develop the taxonomy of social media indicators for disaster response involved a systematic extraction of information and indicators from both grey and academic literature. The grey literature comprised official documentation from humanitarian organisations, including the Emergency Plan of Action documents (IFRC 2018a, 2018b), Disaster Emergency Needs Assessment Reports (Government of Malawi 2015, 2019), Flood Response Plans (FAO 2020; ShelterCluster Org 2017), Impact Assessment Reports (Honaiara City Council 2018; Myanmar Ministry of Agriculture and Irrigation et al. 2015) and Flood Assessment Reports (United Nations Development Programme 2023; Humanitarian Response 2019). Data sets from key studies are also examined to provide understanding of current taxonomies that use social media information (Abavisani et al. 2020; Liu et al. 2019; Nair, Ramya & Sivakumar 2017; Olteanu, Vieweg & Castillo 2015; Santoso 2019; Stowe et al. 2016; Temnikova et al. 2015). Indicators are retrieved from both academic and grey literature sources, which are then filtered to a more manageable and focused list by taking a systematic approach to identify redundancies. Our consolidation process involved the following:

- *Cross-referencing analysis:* We analysed all indicators across all documents and data sets to discern patterns of overlap.
- *Frequency-based selection:* A consolidated list of indicators is developed from the most frequently cited ones across the reports and data sets, signalling their widespread applicability and relevance in disaster response.
- *Disaster-specific inclusion:* Simultaneously, we include the unique set of indicators that are specific to different types of disasters (floods, droughts and heatwaves).

A curated list of social media indicators is developed to represent a balance in both breadth and specificity of information needs by those managing disaster response. This list serves as the foundational framework for the participatory-driven approach, which was further conducted in Ghana and Mauritius for assessing and refining the taxonomy according to the survey results.

### Stakeholder sampling and recruitment strategies

We employed a purposive sampling strategy with an inclusion criterion to ensure stakeholder diversity while addressing potential sampling bias. Stakeholders were defined as individuals with direct professional involvement in disaster management and research, as well as non-governmental organisations (NGOs) and community organisations relevant for humanitarian response. This inclusive definition ensured an initial recruitment targeted at four stakeholder categories: (1) government agencies responsible for disaster response; (2) non-governmental organisations involved in humanitarian assistance; (3) technical experts on disaster research or management; and (4) community organisations with local knowledge. In Ghana and Mauritius, a total of 79 stakeholders were initially contacted through professional networks, governmental databases and NGOs. The invitation sent by e-mail includes the link to the questionnaire under the Redcap platform, with consent recorded before the beginning of the online survey. Firstly, stakeholders read a participant information sheet containing details about the project’s purpose, why they were chosen to take part, options to take part or not, and the importance of collecting data about the use of social media indicators for disaster response, including what happens with the results of the survey. Upon a first round of invitations distributed to our existing networks in Mauritius and Ghana, the number of responses was low. Hence, the snowball method was applied to grow the list of participants, expanding the list to 142 potential participants. An explanatory video was developed and shared with all participants being recruited, highlighting the reasons for data collection and its benefits for potential future improvements of machine learning tools. Both notably increased participation in both locations, which counted with a final participation of 104 respondents (73% response rate).

Despite the multilingual contexts of both Ghana and Mauritius, our stakeholder recruitment and survey administration occurred primarily in English, potentially excluding perspectives from non-English-speaking disaster management practitioners and community members. In Ghana, where Twi, Ga and Ewe are widely spoken, and in Mauritius, where French and Mauritian Creole are prevalent, this language constraint may have systematically excluded some local knowledge and priorities. In addition, our stakeholder recruitment through professional networks may have introduced cultural biases towards Western-educated professionals and urban-based organisations, potentially underrepresenting rural and traditional community perspectives on disaster response priorities.

### Survey development and structure

Our survey design was informed by several key theoretical frameworks. Firstly, we incorporated elements from the Technology-Organization-Environment (TOE) framework (Stieglitz et al. 2018), which helped structure questions around platform use and disaster contexts that influence social media adoption or not. The concept of ‘actionability’ (Kruspe et al. 2019) was also central to our survey design. Rather than just assessing whether participants used social media during disasters, we sought to understand how different stakeholders determined what information was relevant and useful for their specific needs during crisis events. The survey comprised seven sections: (1) organisational profile and social media platform usage, including organisation type and primary social media platforms used; (2) disaster experience history, covering types of disasters dealt with and associated social and/or physical, economic, and environmental and/or cultural losses; (3) information needs during disasters (Appendix 1 Box 1-A1), assessing likelihood of various requests including food/water, shelter, monetary aid, medical assistance, rescue, volunteering, services, disability items, and miscellaneous supplies; (4) damage-related information priorities (Appendix 1 Box 2-A1), covering infrastructure damage reports for water systems, electricity, buildings, vehicles, agriculture, utilities, and pollution; (5) situational awareness information needs (Appendix 1 Box 3-A1), including affected individuals/areas, weather updates, health/disease surveillance, sanitation, public advisories, logistics, safety/security, donations, insurance, and emotional support content; (6) vulnerable population considerations (Appendix 1 Box 4-A1), addressing specific needs related to children, mobility/sensory/cognitively impaired individuals, ill persons, homeless, pregnant women, elderly, socio-economically disadvantaged, indigenous populations, and foreign migrant workers; and (7) **resource assessment,** categorising available resources into own, community, NGO-provided, public, and various capital types (human, social, physical, natural).

### Ethical considerations

The research adhered to comprehensive ethical standards with approval obtained from institutional review boards in all participating countries: University of Ghana Ethics Committee (Reference: ECH 123/20-21), University of Mauritius Research Ethics Committee (Reference: UM-REC-047-2021) and UK institutional approval (Reference: QMUL-HREC-2021-3847). Participant protection measures were implemented throughout the study, beginning with informed consent procedures where all participants received detailed information sheets in English and local languages, explaining study purposes, voluntary participation, data usage and withdrawal rights. Data privacy was ensured through the separation of personal identifiers from responses using unique codes, with all survey responses duly anonymised at the outset. Voluntary participation was emphasised, allowing participants to withdraw at any time without consequence through clearly defined withdrawal procedures, with survey data to be retained for 5 years on secure university servers before permanent deletion.

## Results and analysis

**FIGURE 1:**
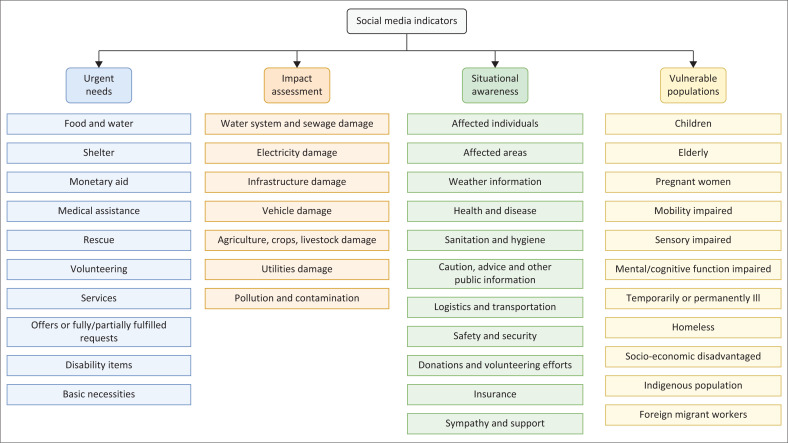
Information needs taxonomy: High-level classes and social media indicators.

A total of 113 indicators were initially identified, representing a broad spectrum of information needs. By cross-referencing and identifying overlap, we consolidated 23 key indicators that are common to multiple reports relevant to the African context. We also identified 16 unique indicators essential for addressing specific types of disasters – floods, droughts and heatwaves. The taxonomy has a total of 39 indicators clustered into four high-level groups: ‘urgent needs’, ‘impact assessment’, ‘situational awareness’ and ‘vulnerable population’. Indicators within each category shared thematic consistency and were frequently interlinked in the literature, reflecting a collective understanding of the core dimensions of disaster response. The urgent needs category, comprising 10 indicators, highlights the consensus in humanitarian reports, such as the Emergency Plan of Action and Needs Assessment, on the priority of addressing life-saving essentials such as shelter, food, water and medical aid. The impact assessment category, with 7 indicators, underscores the importance of real-time information to understand the scope and progression of a crisis, which is essential for effective coordination and response efforts that rely on accurate and timely data. Situational awareness category, consisting of 11 indicators, is crucial for comprehending the consequences of a disaster, as emphasised in Impact Assessment and Flood Response Plans, aiding in both immediate relief efforts and the strategic allocation of resources for long-term recovery. Lastly, the Vulnerable Populations category, also with 11 indicators, focuses on identifying and assisting groups disproportionately affected by disasters, such as the elderly, children and individuals with disabilities, as emphasised in related humanitarian reports. Figure 1 offers a detailed visualisation of the indicators within our taxonomy, aligned with the four distinct categories.

The ‘urgent needs’ category identifies the most immediate and pressing requirements of those affected by a disaster. This includes necessities such as food, water and shelter, as well as specific needs such as medical assistance, rescue operations and volunteering services. By capturing this information through social media, responders can quickly understand and address these critical needs, which are vital for survival and recovery in the immediate aftermath of a disaster. This rapid identification and response to urgent needs can positively impact the effectiveness of the disaster response efforts. Table 1 shows the list of indicators relevant to this category and their definition.

**TABLE 1:** Social media indicators for identifying urgent needs during a disaster.

Urgent needs	Indicator definition
Food and water	Requests for food items, drinking water, etc.
Shelter	Requests for shelter, camp, and accommodation
Monetary aid	Requests for financial assistance
Medical assistance	Requests for medical treatment and assistance for injured/sick people
Rescue	Miscellaneous rescue requests such as finding missing and trapped people
Volunteering	Requests for volunteers to help with response activities such as aid delivery and rescue operations, or availability and willingness to offer volunteering services
Services	Requests for essential services such as electricity, telephone and internet
Offers, fully/partially fulfilled requests	All kinds of offers and all kinds of requests that are fully or partially fulfilled
Disability	Requests for mobility impaired, sensory impaired, wheelchair users, those with injuries requiring assistance such as crutches, individuals who are confined to bed and unable to move, and those who exhibit reduced mobility and move at a lower pace
Basic needs	Requests on miscellaneous items such as blankets, mattresses, mosquito nets, basic household utensils and hygiene items

The second category focuses on using social media for rapid disaster ‘impact assessment’, which is a challenging task for traditional on-ground methods because of time constraints, high cost and risk factors, especially in remote areas. Social media provides real-time, comprehensive insights into local community impacts, offering advantages over conventional methods. Table 2 has seven specific social media indicators, which leverage the immediacy and breadth of social media data to enhance the understanding of the impacts of disasters.

**TABLE 2:** Social media indicators for rapid impact assessment during a disaster.

Urgent needs	Indicator definition
Water system and sewage damage	Reports related to the damage of water systems, dams, rivers, reservoirs, sewage systems, etc.
Electricity damage	Reports related to electricity infrastructure damage, power outages and disruption
Infrastructure damage	Reports related to the damage of buildings (hospitals, houses, schools), roads, bridges and coastal structures; flooded roads; submerged buildings; etc.
Vehicle damage	Reports related to cars damaged, collapse of cranes, trains damaged and other transport-related damages
Agriculture, crops, livestock damage	Reports related to the damage of crops, agriculture, fisheries, natural resources and habitats, irrigation, soil and livestock
Utilities damage	Reports related to the damage of utilities such as telecommunication infrastructure, mobile and landline networks, internet and data centres
Pollution and contamination	Reports related to pollution, contamination and other toxic chemicals in the environment (oceans, soils, air)

The third category is related to the general ‘situational awareness’ of the disaster, which encompasses various topics related to the disaster-affected environment, such as disease outbreaks, safety hazards, weather updates and sanitation issues. This category also includes indicators related to individuals, such as insurance coverage issues, transportation difficulties and family members lost. A total of 11 indicators have been identified in the ‘situational awareness’ category in Table 3.

**TABLE 3:** Social media indicators for improving situational awareness in a disaster.

Urgent needs	Indicator definition
Affected individuals	Reports of injured, dead, missing, found, trapped or homeless people
Affected areas	Reports of affected, damaged roads, streets, cities, states, neighbourhoods, etc.
Weather information	Updates about the weather, forecasts, predictions, water and rainfall information, information of the path, wind and rain and forecast of the trend
Health and disease	Reports related to disease surveillance, and mental, physical or emotional well-being
Sanitation and hygiene	Reports related to the access to potable water, management of waste and sewage, and the presence of sanitary amenities
Caution, advice and other public information	Warnings issued or lifted, instructions to handle certain situations, emergency measures, perceived risks of future flood or other public and official announcements
Logistics and transport	Reports related to delivery and storage of good and supplies
Safety and security	Reports related to safety and security measures, protection of individuals and assets from harm such as violence or theft
Donations and volunteering efforts	Ongoing or completed volunteering efforts, status of donations, and services needed or offered by volunteers or professionals
Insurance	Reports about insurance affordability, availability, claims and other general information related to insurance
Sympathy and support	Covers emotional support, thoughts and prayers

The last category pertains to the identification of ‘vulnerable populations’ during a disaster, where it is crucial to identify and locate individuals who may require additional support and assistance because of their unique circumstances or conditions. This may include the elderly, pregnant women, people with disabilities and other marginalised or vulnerable groups. Table 4 presents eleven indicators covering different types of vulnerable groups.

**TABLE 4:** Social media indicators for improving identification of vulnerable groups.

Vulnerable population	Indicator definition
Children	Reports about children affected/missing/lost in general, schools, etc.
Elderly	Reports about affected elderly people who may require assistance
Pregnant women	Reports about pregnant women who may require assistance
Mobility impaired	Reports detailing individuals who are unable to walk or can only walk short distances, those who require assistance for walking and cases of immobility
Sensory impaired	Reports about people with inability to see, partial ability to see or blindness
Mental/cognitive function Impaired	Reports about individuals with developmental impairments; those requiring clinical psychiatric care; and persons with learning disabilities or challenges related to psychological, emotional or behavioural issues
Temporarily or permanently ill	Reports about persons in need of consistent medical care, such as dialysis, oxygen therapy or a continuous medication regimen, or those suffering from a chronic disease
Homeless	Reports about individuals or families lacking shelter or a place for habitation
Socio-economic disadvantaged	Reports about individuals or groups of people disproportionately affected by the crisis because of their socio-economic status
Indigenous population	Reports about members of indigenous communities who have unique cultural, linguistic and historical backgrounds
Foreign migrant workers	Reports about foreign individuals who may require additional support because of language barriers, limited access to resources or legal status concerns

**FIGURE 2:**
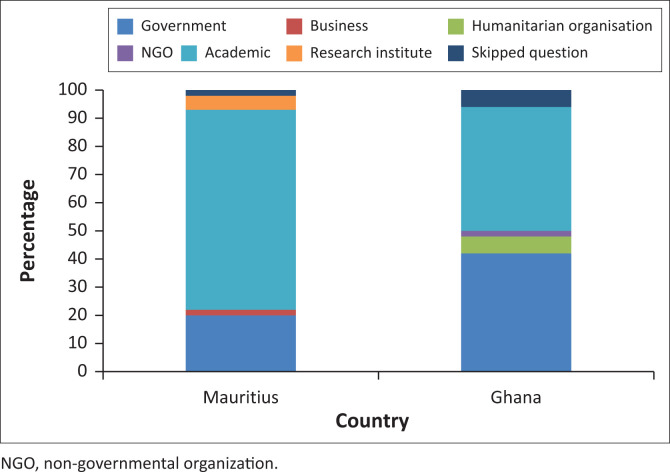
Stakeholder groups engaging with the survey in Ghana and Mauritius.

A total of 104 participants engaged with the survey, mostly coming from governmental bodies and academia (Figure 2). From this total, 46% of the responses are from Ghana and 54% are from Mauritius. Because of missing information in some cases, a total of 45 completed answers are analysed for Ghana and 55 completed answers are analysed for Mauritius. All subsequent analysis is based on these completed responses.

Ghana demonstrates a more heterogeneous stakeholder composition, with representation spanning government, business, humanitarian organisations, NGOs, academic institutions and research institutes. This diversity offers several analytical advantages because of varied operational experiences, priorities and constraints that enrich the evaluation of social media indicators for disaster response. Business and humanitarian organisation representatives contribute practical implementation insights rooted in real-world feasibility and resource constraints, while the mix of government and non-government actors creates important diversity of views and interests. In contrast, Mauritius’ academic-heavy composition creates distinct interpretative considerations, as academic respondents may prioritise indicators based on theoretical research evidence rather than operational constraints. When diverse stakeholders all converge on specific indicators, it suggests these measures possess several critical qualities, including that they might be technically feasible across different organisational capacities, operationally practical for diverse implementation contexts and strategically valuable to serve beyond sector-specific interests.

### Awareness of risk profile

**FIGURE 3:**
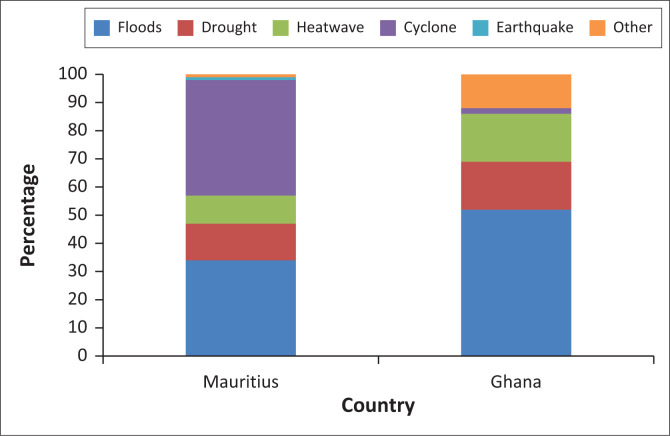
Disaster types affecting stakeholders in Ghana and Mauritius.

Firstly, we asked participants to identify the types of disasters they most frequently manage or experience in their locality (Figure 3). The data reveal that both nations face a variety of disasters, highlighting stakeholders’ broad awareness of diverse threats. This awareness is critical in guiding effective disaster management and preparedness strategies.

In Ghana, floods are the predominant disaster (58%), followed by droughts and heatwaves (each at 17%). In Mauritius, cyclones are the most frequent (41%), with floods close behind (34%), while droughts and heatwaves make up 13% and 10%, respectively (Figure 3). While both countries demonstrate multi-hazard awareness, their primary disaster profiles reflect their distinct geographical characteristics. Ghana’s inland position makes it more susceptible to drought and heat-related events (17% each), while Mauritius’ island status exposes it to cyclones (41%) and associated weather-related hazards. However, both countries share a significant vulnerability to flooding (58% in Ghana, 34% in Mauritius), indicating a common challenge. Sudden onset events such as flash floods require real-time crisis communication indicators.

### Social media uses

**FIGURE 4:**
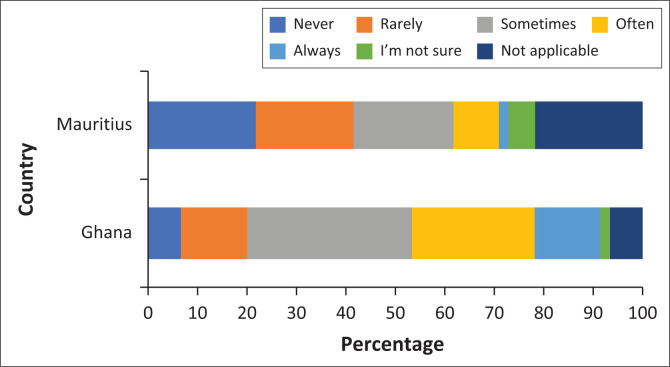
Usefulness of social media according to Ghana and Mauritius stakeholders.

We explore the participants’ outlook on the usefulness of social media for their respective job functions, where a single selection was required from the user. Figure 4 shows a varying pattern in both countries. In Ghana, ‘sometimes’ and ‘often’ selections comprise more than half of the total responses. In contrast, Mauritius had a higher proportion of ‘Not Applicable’ responses, with the remaining responses belonging mostly to ‘Never’, ‘Rarely’ and ‘Sometimes’. This finding suggests that Ghanaians have a more positive outlook towards the role of social media in their professional work as compared to the people of Mauritius.

**FIGURE 5:**
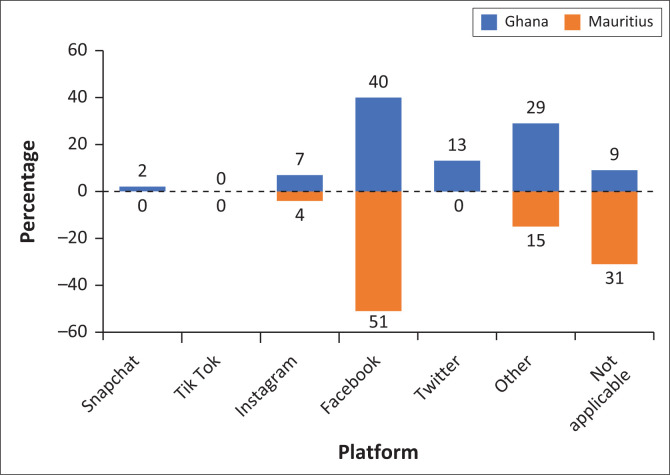
Social media platform used by stakeholders in Ghana and Mauritius.

Figure 5 reveals that Facebook is the primary platform used in both nations. Other commonly used social media platforms include YouTube, email apps, WhatsApp, Telegram and Zoom. This insight is critical for designing AI systems to collect data effectively from social media in disaster contexts. As different platforms may capture varying types of information and allow for different engagement levels, understanding the platform’s preferred use in each country allows for more targeted data collection strategies. Issues in accessing such data will arise depending on the platform rules. Facebook, for example, has reduced third-party access to its data, limiting access to personal posts, comments and certain group interactions, which can hinder the collection of real-time, ground-level insights during a disaster. This data accessibility crisis affects both Ghana and Mauritius equally, as they rely on the same platforms that have similar constraints in accessing citizen-generated disaster information. While academic literature sometimes assumes data availability for social media disaster response, there are rising issues to access the data, so both countries could benefit from developing dedicated emergency communication channels that citizens can voluntarily use during disasters, negotiating emergency data access agreements with platforms, combining limited social media data with traditional communication methods and establishing local volunteer networks to bridge the social media data gap.

### Social media indicators: Nation-specific and category-based

To understand the information needs in the context of disaster response in Ghana and Mauritius, we asked the stakeholders to attach a value between 1 and 5 to each indicator, with 1 being an extremely unlikely indicator and 5 being an extremely likely indicator to be used to help responders. Upon disregarding the ‘Not Relevant’, an average rating was calculated for each indicator. The latter considers the number of responses for each Likert scale category (extremely unlikely, unlikely, neutral, likely, extremely likely) and assigns increasing weights to subsequent classes (1, 2, 3, 4, 5). An example is shown in Table 5 and Table 6 for the ‘Urgent Need’ indicators rated by the stakeholders in Mauritius and Ghana, respectively.

**TABLE 5:** Example of average rating calculation for ‘urgent need’ indicators (Mauritius).

Indicator	Extremely unlikely	Unlikely	Neutral	Likely	Extremely likely	Sum of weights	Sum of responses	Average rating
Food and water	4	1	4	11	9	107	29	3.69
Shelter	1	3	5	7	8	90	24	3.75
Monetary aid	0	0	7	9	10	107	26	4.12
Medical assistance	0	3	6	8	8	96	25	3.84
Rescue	0	4	5	6	8	87	23	3.78
Volunteering	1	4	7	11	5	99	28	3.54
Services	3	3	4	9	9	102	28	3.64
Offers or fully/partially fulfilled	0	3	14	9	2	94	28	3.36
Disability item	0	6	9	6	4	83	25	3.32
Basic necessities	1	2	7	8	10	108	28	3.60

**TABLE 6:** Average rating calculation for ‘urgent need’ indicators (Ghana).

Indicator	Extremely unlikely	Unlikely	Neutral	Likely	Extremely likely	Sum of weights	Sum of responses	Average rating
Food and water	3	2	3	8	16	128	32	4.00
Shelter	2	0	8	12	10	124	32	3.88
Monetary aid	2	2	3	7	18	133	32	4.16
Medical assistance	3	1	2	14	15	142	35	4.06
Rescue	1	4	7	10	12	130	34	3.82
Volunteering	0	2	9	13	9	128	33	3.88
Services	1	3	5	11	13	131	33	3.97
Offers or fully/partially fulfilled	1	4	9	13	9	133	36	3.69
Disability item	0	1	7	14	9	124	31	4.00
Basic necessities	2	2	1	7	19	132	31	4.26

With respect to the urgent needs of the population during and post-disaster event, when a disaster strikes Ghana, social media reports that are classified as ‘Basic Necessities’ should be prioritised. These reports will typically contain information on basic items for survival. Thus, when crisis responders are allocating resources and donations, they should ensure that they are able to meet the demands of the population. Following this, ‘Monetary Aid’ and ‘Medical Assistance’ are the next most important factors that crisis responders should consider when planning a disaster response. While ‘Monetary Aid’ is the second highly ranked indicator in Ghana, it is the first indicator prioritised for Mauritius. The affected people in both locations face various challenges, including limited financial resources/assistance during crises. Following this, ‘Basic Necessities’ and ‘Medical Assistance’ requests are the next most important needs that should be prioritised in Mauritius. Other urgent needs that emerge in the top 5 list for Ghana include ‘Food and Water’ tied with ‘Disability Item’ and ‘Services’, whereas for Mauritius, it is ‘Rescue’ and ‘Shelter’.

To determine the relevance of the ‘Food and Water’ indicator, we calculated a weighted score based on the responses across all rating categories. Each response category was assigned a weight from 1 to 5, with 1 representing ‘Extremely Unlikely’ and 5 representing ‘Extremely Likely’. For ‘Food and Water’, we had 4 responses marked as ‘Extremely Unlikely’, each with a weight of 1, yielding a weighted score of 4. One response was marked as ‘Unlikely’ (weight of 2), giving a score of 2. Four responses were ‘Neutral’ (weight of 3), adding up to 12. Eleven responses were ‘Likely’ (weight of 4), giving 44, and nine responses were ‘Extremely Likely’ (weight of 5), totalling 45. Summing these values, we reached a total weighted score of 107 for the ‘Food and Water’ indicator. To obtain the average rating, we divided the total weighted score (107) by the number of responses (29), resulting in an average score of 3.69. This same method was applied to all social media indicators in both Ghana and Mauritius (Appendix 1 Table 1-A1, Table 2-A1, Table 3-A1), with the average ratings for each indicator displayed in Table 7.

**TABLE 7:** Average rating of all social media indicators in selected case studies.

Category	Indicator	Ghana	Mauritius
Urgent needs	Food and water	4.00	3.69
	Shelter	3.88	3.75
	Monetary aid	4.16	4.12
	Medical assistance	4.06	3.84
	Rescue	3.82	3.78
	Volunteering	3.88	3.54
	Services	3.97	3.64
	Offers or fully/partially fulfilled	3.69	3.36
	Disability item	4.00	3.32
	Necessities	4.26	3.86
Impact assessment	Water system and sewage damage	4.07	3.89
	Electricity damage	3.96	4.19
	Infrastructure damage	4.07	4.07
	Vehicle damage	3.82	3.65
	Agriculture, crops, livestock damage	4.26	4.15
	Utilities damage	3.59	3.97
	Pollution and contamination	3.81	3.67
Situational awareness	Affected individuals	4.29	3.60
	Affected areas	4.05	4.00
	Weather information	3.63	4.20
	Health and disease	3.91	3.96
	Sanitation and hygiene	3.70	3.85
	Caution, advice and other public information	3.65	4.20
	Logistics and transportation	3.57	3.46
	Safety and security	3.87	3.69
	Donations and volunteering efforts	4.22	3.85
	Insurance	2.96	3.40
	Sympathy and support	3.70	3.54
Vulnerable populations	Children	4.58	1.55
	Mobility impaired	3.67	1.65
	Sensory impaired	3.69	1.76
	Mental/cognitive function impaired	4.00	1.63
	Temporarily or permanently ill	3.85	1.65
	Homeless	4.25	1.72
	Pregnant women	3.92	1.84
	Elderly	4.00	1.81
	Socio-economic disadvantaged	3.92	1.56
	Indigenous population	3.55	1.85
	Foreign migrant workers	3.36	1.44

**FIGURE 6:**
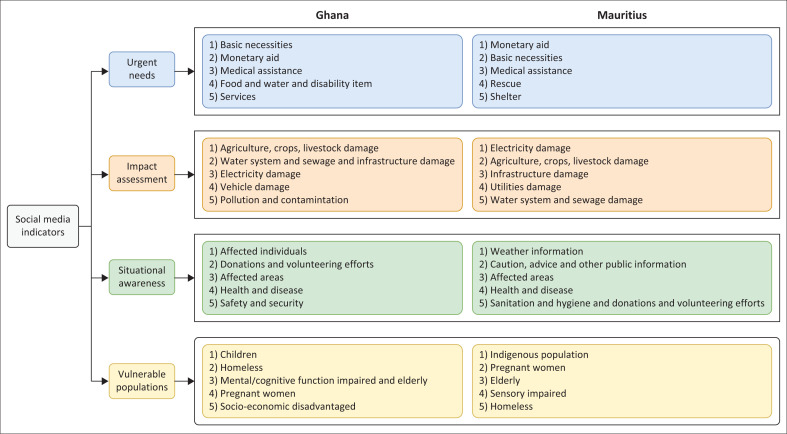
Top five priority social media indicators for Ghana and Mauritius.

One of the primary objectives of the survey is to examine priority information needs within each high-level category during specific disaster events and to identify potential similarities or differences between Ghana and Mauritius. To establish the priority ranking of information needs within each category, the social media indicators were ordered based on their average ratings in descending order, as presented in Table 7. Figure 6 illustrates the top five prioritised indicators for both Ghana and Mauritius across the four key categories, namely, urgent needs, impact assessment, situational awareness and vulnerable populations. This comparative analysis enables a deeper understanding of how information priorities may vary between different national contexts while revealing common patterns in disaster-related information requirements.

When comparing the top 5 indicators, there is little overlap between the nations, with only ‘Donations and Volunteering Efforts’ and ‘Affected Areas’ being common. Ghana prioritises indicators such as affected individuals, donations and volunteering efforts, affected areas, health and disease, and safety and security, emphasising the immediate human impact and safety concerns in disaster scenarios. This focus indicates Ghana’s need to address direct impacts on people and community well-being promptly. Mauritius, on the other hand, places higher importance on indicators such as weather information, public advice and caution information, affected areas, health and disease, and sanitation and hygiene, along with donations and volunteering efforts. The prioritisation of weather information and public advisories suggests that Mauritius focuses on preventive measures and real-time updates, which are critical for an island nation facing risks such as cyclones and other weather-related disasters.

### Impact assessment: Infrastructure vulnerabilities parallels

Both countries show alignment in impact assessment priorities, with four of the top five indicators being identical: agriculture/crops/livestock damage, electricity damage, water systems/sewage damage and infrastructure damage. This convergence indicates shared vulnerabilities that reflect common developmental challenges in emerging economies, recognising agriculture as economically critical (4.26 Ghana, 4.15 Mauritius), acknowledging that power grid vulnerabilities affect both nations similarly with electricity infrastructure priorities (3.96 Ghana, 4.19 Mauritius), identifying that essential water and sewage systems face threats (4.07 Ghana, 3.89 Mauritius) and showing identical ratings for general infrastructure damage (4.07 for both countries). Additional indicators in Ghana that show up in the top 5 list include ‘Vehicle Damage’ and ‘Pollution and Contamination’, whereas in Mauritius, an additional indicator of ‘Utilities Damage’ is included. While these commonalities dominate their priorities, each country also reflects unique contextual needs. Ghana’s inclusion of vehicle damage highlights transportation infrastructure damages, while Mauritius’ focus on utilities damage aligns with the small island developing state’s connectivity challenges. The convergence we observed in infrastructure damage indicators could likely reflect the influence of international donor priorities and reporting requirements rather than local consensus, highlighting how taxonomies can potentially mask rather than address differences in community needs.

### Situational awareness: Divergent strategies with common goals

While showing the least overlap in top priorities, both countries share fundamental concerns about ‘Donations and Volunteering Efforts’ and ‘Affected Areas’, indicating common needs for resource coordination and geographic impact assessment. Overall, Ghana prioritises immediate human impact indicators (Affected Individuals: 4.29, Health and Disease: 3.91, Safety and Security: 3.87), suggesting a response strategy focused on direct population welfare and immediate safety concerns. Mauritius emphasises anticipatory measures (Weather Information: 4.20, Caution/Advice: 4.20), reflecting island nation adaptation strategies where early warning and prevention are crucial for survival. Ghana’s human-centred focus offers important lessons about prioritising immediate population welfare and safety concerns during disaster response. These divergent approaches are complementary and offer valuable lessons for cross-national learning.

### Vulnerable populations: Different priorities for common concerns

This category reveals divergence between countries, yet underlying patterns suggest different approaches to the same fundamental goal of protecting society’s most vulnerable members. Ghana consistently rates all vulnerable population indicators between 3.36 and 4.58, with particularly high scores for children (4.58), homeless populations (4.25) and mentally/cognitively impaired individuals (4.00). This broad, high-rating pattern suggests a comprehensive social protection philosophy. Mauritius shows more selective prioritisation, with significantly lower ratings (1.44-1.85) but targeted emphasis on indigenous populations (1.85) and pregnant women (1.84). This focused approach may reflect resource constraints or specific demographic priorities. The analysis of vulnerability indicators is essential in disaster management, as certain groups – such as women, children and residents of informal settlements – are disproportionately impacted by disasters.

In Ghana, ‘Children’ are the top priority among vulnerable groups, highlighting the need for crisis managers to focus their interventions on ensuring the safety, well-being and protection of young individuals. In contrast, in Mauritius, ‘Indigenous Populations’ are the primary focus, indicating that response strategies should prioritise safeguarding the unique cultural and community needs of these groups during crises. Both countries share a common emphasis on pregnant women, the elderly and homeless people, who require tailored support to manage their specific vulnerabilities. Crisis responders should prioritise medical assistance for pregnant women, provide essential support tools (such as wheelchairs) for elderly individuals and ensure safe shelter options for homeless populations to mitigate their exposure to disaster impacts. Furthermore, Ghana places a higher priority on addressing the needs of mentally impaired and socio-economically disadvantaged populations, recognising the distinct challenges they face during disasters. This calls for targeted support strategies, such as providing mental health resources and addressing socio-economic vulnerabilities to enhance resilience. Conversely, in Mauritius, there is a significant focus on sensory-impaired individuals, necessitating accessible communication methods to ensure they receive critical information and assistance during emergencies. These variations in vulnerability priorities underscore the need for more localised disaster response strategies that account for the unique demographics and specific needs within each country.

## Discussions, limitations and future research

This study developed a collaborative taxonomy of social media indicators for disaster response systems through extensive literature review and stakeholder consultation across Ghana and Mauritius. The prioritised indicators reveal regional variations in disaster response information needs, with insights for both taxonomy development and future machine learning applications. Ghana’s emphasis on security against property theft, gender-based violence prevention and community safety monitoring reflects the complex social dynamics that emerge during disaster response phases. These concerns align with broader research findings about how disasters have highly stratified effects affecting most adversely those who are already disadvantaged, with recovery being delayed because of corruption, profiteering or inadequate governance leading to second-order disasters. Mauritius’ focus on vulnerable age groups, transportation access and safe logistics coordination reflects the unique challenges of small island developing states, where infrastructure constraints and geographic isolation amplify disaster impacts. The collaborative methodology employed here provides a replicable framework for exploring disaster response taxonomies adapted to local contexts, though it requires significant stakeholder engagement and continuous knowledge building. The latter and other key limitations of this study inform our discussion of future research pathways.

The cross-sectional nature of our study captured stakeholder opinions at a single point in time without accounting for how these priorities might change based on recent disaster experiences or changing institutional contexts. Our analytical approach relied on simple average ratings without sophisticated statistical analysis to control for potential confounding variables such as organisational type, individual experience level or specific disaster exposure history. Furthermore, our sample size within each stakeholder category (government, NGO, academic) was relatively small, limiting our ability to conduct robust subgroup analyses. Also, our focus on only two countries, while providing valuable comparative insights, limits the generalisability of regional patterns. Future work should expand to include a broader range of geographic contexts, disaster types and cultural settings to develop a more comprehensive understanding of taxonomy variation patterns.

The reliance on English-language literature and primarily English-speaking stakeholders represents another significant limitation. Disaster communication often occurs in local languages with cultural nuances that may not translate directly to English-based taxonomies. Future research should prioritise multilingual taxonomy development and explore how language-specific communication patterns influence indicator relevance and interpretation. The multilingual and multicultural nature of both Ghana and Mauritius presents unique opportunities and challenges for social media analytics deployment. In Ghana, the linguistic diversity encompassing English, Twi, Ga and Ewe requires sophisticated natural language processing capabilities that can handle code-switching patterns common in Ghanaian social media use. Similarly, Mauritius’ trilingual environment of English, French and Mauritian Creole necessitates models capable of processing multilingual content while maintaining semantic understanding across languages.

The temporal aspect of disaster response was not fully captured in our static taxonomy. Social media information needs evolve throughout disaster cycles – from preparedness through response to recovery. Future work could investigate dynamic taxonomies that can adapt to different disaster phases and develop machine learning systems capable of adjusting their classification priorities accordingly. Moreover, while our taxonomy focused on text, recent multimodal deep learning approaches combining textual and visual data achieve over 91% accuracy in disaster assessment. Future research should extend our participatory methodology to develop region-specific taxonomies capable of capturing social media images, videos and audio content.

**FIGURE 7:**
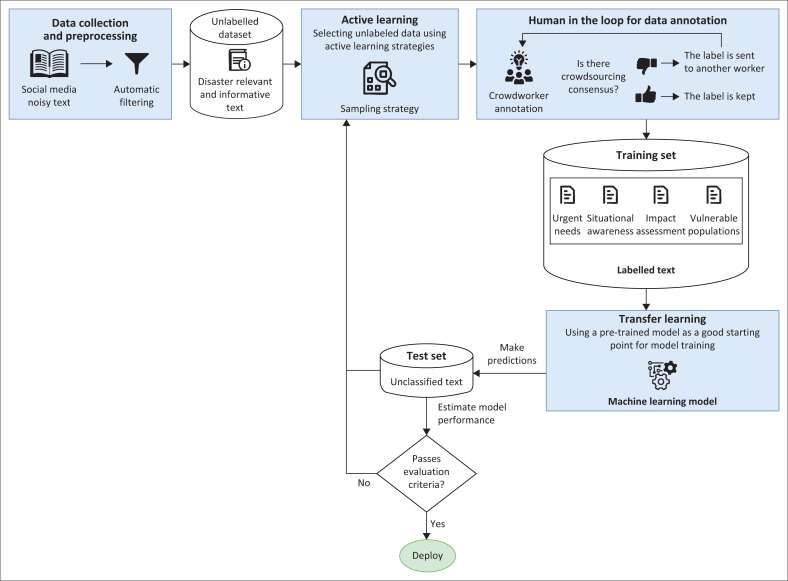
Workflow for training machine learning classifiers based on the developed taxonomy.

Finally, this study lacks systematic validation of the taxonomy’s empirical effectiveness in real-world disaster contexts. While our participatory approach ensured stakeholder input in priority ranking, we did not test how the resulting taxonomy improves information extraction from social media data during disaster events in these locations. This study’s approach prioritised stakeholder input over empirical validation, creating methodological limitations. In future research, we aim to verify whether our stakeholder-prioritised indicators correspond to the types of information people share during crises. Our categories may include theoretically important but practically rare information types or conversely, may miss emerging communication patterns not captured in formal humanitarian documents. Despite limitations, the differences in priorities within each high-class category of social media indicators suggest that it is likely worth exploring region-specific machine learning models in the future. In order to train machine learning classifiers that can classify text based on our taxonomy, we developed an illustrative workflow in Figure 7 to serve as one of many pathways for exploring the use of machine learning methods for the collection and processing of social media data through localised approach.

Our proposed social media data processing workflow for disasters involves six key steps, namely, *data collection* from local social media platforms; *active learning* to select the most informative samples using techniques such as uncertainty sampling; *human annotation* where crowdworkers label data with multiple agreement requirements and fair working conditions; *transfer learning* using pre-trained humanitarian classification models; *model testing* to evaluate accuracy on unclassified messages, and iterative; and *improvement* through batch-mode learning until deployment-ready accuracy is achieved. This human-in-the-loop approach emphasises quality control through crowdworker consensus, continuous model enhancement through iterative training batches and adaptability to local stakeholder needs, while addressing challenges around scalability and real-time deployment during actual disaster events.

The technical architecture for regional adaptation requires sophisticated transfer learning approaches that can leverage pre-trained multilingual models while incorporating region-specific context. Many approaches for crisis social message detection employ Word2vec and other embeddings, with crisis-specific versions showing promise, while recent developments in BERT embeddings and their various offshoots have become very popular, including crisis-specific versions. For Ghana and Mauritius, this would mean starting with multilingual models like mBERT or XLM-R and fine-tuning them on regional disaster data sets while incorporating cultural context embeddings that capture local communication patterns and disaster-related terminology.

The evaluation frameworks must also be adapted to reflect regional priorities and operational contexts. Traditional metrics focusing on general informativeness may not capture the nuanced requirements of local emergency management agencies. Emergency management agencies acknowledge the benefits of extracting information from social media, for example, gaining better situational awareness, and appreciate the possibility of monitoring social media in real time, thus always being informed about the current position of affected citizens. For Ghana, this means developing metrics that can accurately assess the detection of security incidents and gender-based violence concerns, while for Mauritius, the focus should be on transportation disruption classification and infrastructure status reporting accuracy.

The deployment considerations would benefit from going beyond technical capabilities to encompass integration with existing emergency management systems and community structures. Technical solutions should ideally include crisis managers in the sense-making and information validation process for high acceptance of social media analytics. Emergency management agencies need customisable filtering algorithms to respond effectively to their specific crisis situations. This requires close collaboration with local institutions such as Ghana’s National Disaster Management Organization and Mauritius Meteorological Services to ensure that the adapted taxonomies align with existing operational procedures and information needs.

The proposed human-in-the-loop annotation process, while ensuring quality, presents scalability challenges for rapid deployment during actual disasters. Future research should explore semi-automated annotation techniques that can maintain quality while reducing human annotation requirements. This could include developing confidence measures for automated annotations and identifying when human intervention is most critical. The batch-mode learning approach suggested in our workflow requires further investigation regarding optimal batch sizes and update frequencies during disaster events. Real-time adaptation of classifiers during ongoing disasters presents unique technical challenges that current machine learning frameworks are not specifically designed to address.

## Conclusion

Our research makes three contributions to the field of disaster informatics and social media analytics. Firstly, we demonstrate that universal taxonomies are likely inadequate for addressing regional disaster response needs. Current approaches often fail to account for the ‘use case-dependent actionability’ of information, where what constitutes relevant or actionable information varies based on the specific role of the user and the local context of the disaster. Secondly, we provide empirical evidence of regional variations in disaster information priorities across all four taxonomy categories. While some indicators showed convergence, particularly in infrastructure damage impact assessment categories, others revealed stark differences that would render universal classifiers ineffective. These variations reflect deeper cultural, economic and institutional differences that must be incorporated into future AI system design. Thirdly, we propose a methodological framework for participatory taxonomy development that can help ground future AI systems in local knowledge and stakeholder priorities.

The evidence of regional variations across all taxonomy categories reveals that disaster management community has been building AI systems based on assumptions about information universality that are questionable. Our proposed workflow (Figure 7) provides a technical framework for exploring context-aware classifiers that can adapt to local priorities while maintaining the efficiency of automated processing. This approach being proposed as future research moves beyond static, universal categorisations to create dynamic, context-aware classification systems that capture unique regional priorities while ensuring disaster response effectiveness. This framework emphasises adaptive AI for crisis response that continuously learns from local stakeholder feedback rather than relying on static models. Pre-trained systems must be culturally adapted using regional data sets that reflect local languages, contexts and disaster-specific terminology. Multi-stakeholder participation is essential, ensuring AI tools incorporate diverse perspectives through ongoing feedback loops to maintain relevance and accuracy in emergency situations.

The scalability of regionally adapted taxonomies requires establishing sustainable feedback mechanisms that can continuously improve system performance based on local expert input and changing disaster patterns. A shift from static pre-trained models to more adaptable and flexible machine learning methods is required, with approaches such as domain adaptation and active learning demonstrating that traditional pre-trained models can be utilised in a more interactive fashion and therefore have the potential to better fit to needs of emergency responders. However, the successful adaptation of social media analytics for disaster management in regions such as Ghana and Mauritius requires a holistic approach that combines technical innovation with deep cultural understanding, community engagement and ethical consideration.

The ethical considerations become particularly complex when deploying social media analytics in regions where data protection frameworks may differ from Western standards and where vulnerable populations may face additional risks from data exposure. The status of information shared under extreme conditions should earn greater protections and ethical consideration rather than less, as people’s privacy preferences depend on their circumstances, and their choices shift depending on their situation. This necessitates developing privacy-preserving approaches that can extract actionable insights while protecting individual privacy and preventing potential harm to disaster-affected communities.

Future research should address current limitations through systematic empirical validation using real social media data from Ghana and Mauritius disasters, measuring classification performance improvements against universal taxonomies through controlled experiments. Key methodological enhancements include implementing inter-rater reliability testing with Cohen’s kappa calculations, expanding geographic validation across additional African and Small Island Developing States, and developing multilingual models for code-switching social media patterns. Technical priorities involve creating privacy-preserving analytics frameworks, establishing emergency data access agreements with social media platforms and building institutional capacity through training programmes for local emergency management agencies.

## Appendix 1

BOX 1-A1 Urgent needs questionnaire.

BOX 2-A1 Impact assessment questionnaire.

**TABLE 1-A1:** Average rating calculation for ‘impact assessment’ indicators (Mauritius).

Indicator	Extremely unlikely	Unlikely	Neutral	Likely	Extremely likely	Sum of weights	Sum of responses	Average rating
Water system and sewage damage	2	2	3	11	10	109	28	3.89
Electricity damage	1	0	1	16	9	113	27	4.19
Infrastructure damage	0	1	4	14	8	110	27	4.07
Vehicle damage	1	3	6	10	6	95	26	3.65
Agriculture, crops, livestock damage	0	2	1	15	9	112	27	4.15
Utilities damage	1	1	4	16	8	119	30	3.97
Pollution and contamination	4	2	2	14	8	110	30	3.67

BOX 3-A1 Situational awareness questionnaire.

**TABLE 2-A1:** Average rating calculation for ‘situational awareness’ indicators (Ghana).

Indicator	Extremely unlikely	Unlikely	Neutral	Likely	Extremely likely	Sum of weights	Sum of responses	Average rating
Affected individuals	1	2	1	5	15	103	24	4.29
Affected areas	2	0	1	11	8	89	22	4.05
Weather information	1	4	3	11	5	87	24	3.63
Health and disease	1	2	2	10	7	86	22	3.91
Sanitation and hygiene	1	2	7	6	7	85	23	3.70
Caution, advice and other public information	2	2	5	7	7	84	23	3.65
Logistics and transport	1	4	5	7	6	82	23	3.57
Safety and security	1	2	4	8	8	89	23	3.87
Donations and volunteering efforts	1	0	3	8	11	97	23	4.22
Insurance	5	5	4	6	4	71	24	2.96
Sympathy and support	1	3	3	11	5	85	23	3.70

BOX 4-A1 Vulnerability questionnaire.

**TABLE 3-A1:** Average rating calculation for ‘vulnerable population’ indicators (Ghana).

Indicator	Extremely unlikely	Unlikely	Neutral	Likely	Extremely likely	Sum of weights	Sum of responses	Average rating
Children	0	0	0	5	7	55	12	4.58
Elderly	0	0	3	6	3	48	12	4.00
Pregnant women	0	2	0	8	3	51	13	3.92
Mobility impaired	0	2	2	6	2	44	12	3.67
Sensory impaired	0	2	3	5	3	48	13	3.69
Mental/cognitive function impaired	0	0	3	6	3	48	12	4.00
Temporarily or permanently ill	0	1	3	6	3	50	13	3.85
Homeless	0	0	1	7	4	51	12	4.25
Socio-economic disadvantaged	1	0	1	7	3	47	12	3.92
Indigenous population	0	3	1	5	2	39	11	3.55
Foreign migrant workers	1	2	2	4	2	37	11	3.36
